# Changes in synovial fluid biomarkers and clinical efficacy of intra-articular injections of hyaluronic acid for patients with knee osteoarthritis

**DOI:** 10.1186/s40634-014-0016-7

**Published:** 2014-12-20

**Authors:** Yoshihiro Kusayama, Yasushi Akamatsu, Ken Kumagai, Hideo Kobayashi, Masato Aratake, Tomoyuki Saito

**Affiliations:** Department of Orthopaedic Surgery, Yokohama City University School of Medicine, 3-9 Fukuura, Kanazawa-ku Yokohama, 236-0004 Japan

**Keywords:** Intra-articular hyaluronic acid, Knee osteoarthritis, Biomarker, Viscosity, Synovial fluid, Interleukin-6

## Abstract

**Background:**

The changes in synovial fluid biomarkers after intra-articular injection of hyarulonic acid (IA HA) remain controversial. We investigate the changes in the properties of synovial fluid (SF) and clinical symptoms before the first and fifth IA HA.

**Methods:**

A total of 73 patients (73 knees) with symptomatic knee osteoarthritis were treated with five weekly intra-articular injections of HA and 55 patients (55 knees) were analyzed. The SF total volume, viscosity, and levels of HA, chondroitin 4-sulfate (C4S), chondroitin 6-sulfate (C6S), keratin sulfate, and interleukin (IL)-6 were measured before the first and fifth injections. Clinical evaluations were performed using the American Knee Society score for physician-based outcome measurements and Knee injury and Osteoarthritis Outcome Score for patient-based outcome measurements before the first and fifth injections.

**Results:**

The SF viscosity and levels of HA were significantly increased, and the total SF volume and levels of chondroitin 4-sulfate, chondroitin 6-sulfate, and interleukin-6 were significantly decreased. The physician-based and patient-based outcome scores were improved.

**Conclusions:**

Our findings speculate that HA injections significantly modulate levels of intra articular biomarkers which may indicate beneficial effect for articular cartilage and synovium membrane.

## Background

Knee osteoarthritis (OA) is a common disease in aging people. The characteristics of OA are degeneration and destruction of the articular cartilage, with secondary induction of hydrarthrosis and synovitis by inflammatory cytokines and growth factors from the destroyed cartilage and synovial membrane [1]. In advanced OA, hyaluronic acid (HA) is decreased in the synovial fluid (SF) [2], and the SF viscosity and chondroprotective function are also reduced. These changes cause further destruction of the articular cartilage.

The theoretical basis of intra-articular injection of HA (IA HA) is to improve the joint lubrication and SF viscosity [3]. The efficacy of IA HA is controversial in the past randomized control trials or meta-analysis [4-12].The American Academy of Orthopaedic Surgeons stated that IA HA is no longer recommended in the 2^nd^ edition of their guideline for knee OA treatment published in 2013 [13]. The Osteoarthritis Research Society International commented that the treatment appropriateness of IA HA is uncertain in their guidelines for the management of knee OA [14]. HA drug for intra-articular injection was produced in Japan at 1987, IA HA was still widely used for treatment of knee OA to this day and we have confidence in this therapy. Therefore, the efficacy and safety of IA HA remain controversial between Japan and other countries.

Many biomarkers are reported to be related with this pathogenesis. Some types of biomarkers reflect cartilage degeneration, others reflect inflammation condition of synovium. The destruction of joint cartilage associated with OA degeneration leads to the release of chondroitin 6-sulfate (C6S), chondroitin 4-sulfate (C4S), and keratin sulfate (KS) from proteoglycan molecules into the SF, which can also be measured as biomarkers of joint cartilage turnover [15,16]. We used biomarkers in SF for evaluation of IA HA, because these factors directly reflect the states of the articular cartilage and synovial membrane. Inflammation is one of the factors associated with OA changes [17,18]. Synovitis, which is secondarily induced, is related to joint destruction and OA progression [19]. Inflammatory cytokines are released from the synovial membrane under the synovitis condition. Interleukin-1-β (IL-1β), tumor necrosis factor-α (TNF-α), matrix metalloproteases (MMP-1, MMP-3, MMP13) are known for proinflamatory mediators [20]. IL-6 is also one of the inflammatory cytokines that is especially elevated in rheumatoid arthritis [21,22]. IL-6 is also detected in SF from patients with knee OA, and high levels of IL-6 are associated with the development of OA and pain severity [23-27]. We measured the IL-6 concentrations as a biomarker for inflammatory reactions in knee OA treated by IA HA. The first therapeutic purpose of IA HA is supplementation of the decreased HA concentration and viscosity in SF induced by OA changes, but few researchers have measured the HA concentration and viscosity in SF after IA HA [3,28-30]. We evaluated the HA concentration and viscosity of SF to evaluate the practical changes in the SF properties after IA HA treatment.

We performed five weekly IA HA treatments. We hypothesize that HA has condroprotective effect and inhibitory effect for synovitis, and that biomarkers which indirectly reflect cartilage metabolism and inflammation condition would be changed after IA HA treatment. No studies have measured biomarkers of cartilage degeneration products, inflammation, and viscosity in SF and clinical results at the same time. The main purpose of this study was to investigate the changes in SF viscosity, HA, C4S, C6S, and KS as cartilage degenerative biomarkers, IL-6 as an inflammatory biomarker, and also we assessed clinical symptoms before and after IA HA treatments.

## Methods

### Patients

The study subjects were all patients who fulfilled the American College of Rheumatology criteria for knee OA [31] and visited our outpatient clinic between January 2010 and August 2013. The inclusion criteria for the study were: (1) age above 40 years; (2) radiographic classification of knee OA of Kellgren–Lawrence (K/L) grade 2 or 3 [32]; and (3) hydroarthrosis detected by clinical examination. The exclusion criteria were: (1) previous treatment with IA HA or corticosteroid; (2) opioid or non-steroidal anti-inflammatory drug administration within the previous 6 weeks; (3) knee arthritis caused by rheumatoid arthritis, gout, pseudogout, avascular necrosis, injury, or joint infection; and (4) surgical treatment including arthroscopy or high tibial osteotomy. All patients provided informed consent and agreed to participate in the study. This study was approved by the ethics committee of our hospital. Ethical committee approval number is B1000902020.

### Intra-articular injection and sample collection

Patients were treated with five weekly intra-articular injections of 1% HA (low molecular weight: approximately 900 kDa) solution with a dosage of 2.5 ml/injection (Artz; Seikagaku Corporation, Tokyo, Japan). The SF samples were collected as much as possible through a lateral suprapatellar approach using a 21-gauge needle before each HA injection, and centrifuged at 15,000 × *g* for 15 minutes at room temperature. The supernatants were collected and immediately stored at −80°C until analysis. The biomarkers in SF were analyzed before the first and fifth IA HA treatments.

### Measurement of joint biomarkers

The C6S and C4S levels in SF were measured by high performance liquid chromatography according to the method reported by Shinmei et al. [15]. The SF samples were diluted 10-fold and subjected to a series of digestions with chondroitinase ABC and chondroitinase AC-II (Seikagaku Corporation). The chondroitinase digestions produced the unsaturated disaccharides Δdi-6S and Δdi-4S from the CS chain structures in C6S and C4S, respectively. After ultrafiltration of the digested solutions, the levels of Δdi-6S and Δdi-4S in the filtrates were analyzed, and the area of the peak corresponding to each unsaturated disaccharide was calculated.

The KS levels in SF were measured by high performance liquid chromatography according to the method reported by Yamada et al. [33]. The SF samples were diluted 10-fold and treated with keratanase II (Seikagaku Corporation) for digestion into two disaccharide isomers, β-galactosyl-(1–4)-6-0-sulfo-N-acetylglucosamine (L2) and β-6-0-sulfo-galactosyl-(1–4)-6-0-sulfo-N-acetylglucosamine (L4). The concentrations of these disaccharide isomers were determined and the sum of their levels was considered to be the KS level. The HA levels in SF were measured by the Morgan–Elson method [34]. The SF samples were digested with HAase SD (Seikagaku Corporation), and the concentration of the resulting unsaturated disaccharides was determined as the HA level.

The IL-6 levels in SF were measured by chemiluminescent enzyme immunoassay (CLEIA) using mouse monoclonal antibodies against recombinant IL-6 (Fujirebio Inc., Tokyo, Japan) [35]. The assay format was based on a two-step sandwich CLEIA method. Briefly, 160 μl of SF was added to 50 μl of a suspension of ferrite microparticles coated with a monoclonal antibody against IL-6 as a solid phase and incubated for 10 minutes at 37°C. The particles were separated magnetically and washed, and 250 μl of another monoclonal antibody against IL-6 conjugated with alkaline phosphate was added to the particles. After incubation at 37°C for 10 minutes, the particles were washed again. Subsequently, the substrate solution [AMPPD; 3-(2′-spiroadamantane)-4-methoxy-4-(3″-phosphoryloxy) phenyl1,2-dioxetane disodium] was added at 37°C. After incubation for 5 minutes, the chemiluminescent signals were photon-counted. The assay was performed using a Lumipulse ƒ system (Fujirebio Inc.), which is a fully automated CLEIA analyzer.

### Measurement of SF viscosity

Viscosity measurements were performed using a cone-flat plate-type rotational viscometer in the same way which was reported by the XIV Japanese Pharmacopeia [36]. SF was introduced to fill the gap between a flat disc and a cone forming an angle, α (rad). When either the flat disc or the cone was rotated at a constant angular velocity or constant torque, the torque acting on the disc or cone surface rotated by the viscous flow and the corresponding angular velocity in the steady state were measured. The viscosity of the SF, *η*, was calculated using the following equation:

$$ \eta =\frac{3\alpha }{2\pi R{}^3}\cdot \frac{100\mathrm{T}}{\omega } $$

where *η* is the viscosity of the liquid (mPa · S), π is the circumference/diameter ratio, R is the radius of the cone (cm), α is the angle between the flat disc and the cone (rad), ω is the angular velocity (rad/s), and T is the torque acting on the flat disc or cone surface (10^−7^ N · m).

### Radiographs

A weight-bearing anteroposterior knee radiograph was acquired at the first outpatient clinic visit for assessing inclusion or exclusion criteria. The degree of knee degeneration was classified according to the K/L grade [32]. All patients were graded by two observers (YK and YA) to avoid differences in incomplete radiographic grading. The femorotibial angle was measured by one of the observers (YK).

### Evaluation of clinical symptoms

We examined the clinical symptoms before the first and fifth IA HA treatments. Clinical efficacy was evaluated by the American Knee Society (AKS) score for physician-based outcomes and Knee injury and Osteoarthritis Outcome Score (KOOS) for patient-based outcomes [37,38]. We used the AKS knee and function scores. The KOOS is a questionnaire that covers five patient-related dimensions: pain; other disease-specific symptoms; activities of daily living; sport/recreation functions; and knee-related quality of life. For each question, five alternatives are presented, with a score range from 0 to 4 points. The KOOS questionnaires were filled out by the patients before the first and fifth IA HA treatments.

### Statistical analysis

All statistical analyses were performed using SPSS for Windows (SPSS, Chicago, IL, USA). Data were expressed as mean ± standard deviation. Biomarkers were compared using Wilcoxon’s rank-sum test. For correlation analyses, Spearman’s rank correlation was used. Values of *P <* 0.05 were considered significant.

## Results

### Characteristics of the patients

A total of 73 outpatients (73 knees) with symptomatic knee OA who had indications for IA HA treatment were enrolled in this study. Eighteen patients (18 knees) became dry knee before the five weekly IA HA treatments were completed and they were excluded. Therefore, 55 knees (27 right knees and 28 left knees) were registered. The study subjects comprised 40 females and 15 males with a mean age and body mass index of 66.7 ± 9.0 years and 23.7 ± 2.8 kg/m^2^, respectively. The K/L grades were grade 2 in 34 knees, grade 3 in 21 knees. The mean femorotibial angle was 178.3 ± 3.3°.

### Changes in biomarkers after injections

**Figure 1:**
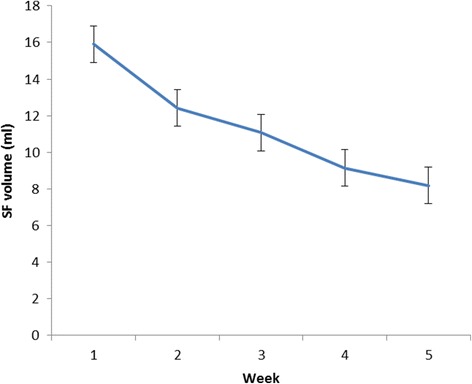
**Time-course changes of mean SF volume before each injection.**

The mean total SF volume, which was 15.9 ml before the first injection and 8.7 ml before the fifth injection, was significantly decreased by the IA HA treatments (*P <* 0.001) (Figure 1). The mean HA level was significantly increased from 1.48 mg/ml to 1.73 mg/ml (*P <* 0.001). The mean C6S and C4S levels were significantly decreased from 62.2 nmol/ml to 49.6 nmol/ml (*P <* 0.05) and from 18.2 nmol/ml to 15.9 nmol/ml, respectively (*P <* 0.05). The mean KS level was not significantly changed from 6.70 μg/ml to 6.18 μg/ml. The mean IL-6 level was significantly decreased from 3458 pg/ml to 486 pg/ml (*P <* 0.05). The mean SF viscosity was significantly increased from 48.6 mPa · S to68.7 mPa · S (*P <* 0.001) (Table 1).

**Table 1:** **Levels of biomarkers and volume in SF before the first HA injection (week 0) and fifth injection (week 5)**

	**Week 0**	**Week 5**	***P*** **value**
HA (mg/ml)	1.48 ± 0.40	1.72 ± 0.36	<0.001
C6S (nmol/ml)	62.2 ± 30.4	49.6 ± 18.4	0.001
C4S (nmol/ml)	18.2 ± 6.3	15.9 ± 4.6	0.001
KS (μg/ml)	6.68 ± 4.25	6.18 ± 3.81	0.172
IL-6 (pg/ml)	3458 ± 9094	486 ± 1294	0.013
Viscosity (mPa · S)	48.1 ± 26.7	67.8 ± 35.2	<0.001
SF (ml)	15.9 ± 12.5	8.2 ± 8.7	<0.001

SF: synovial fluid. HA: hyaluronic acid. C6S: chondroitin 6-sulfate. C4S: chondroitin 4-sulfate. KS: keratin sulfate. IL-6: interleukin-6.

Data are given as mean ± standard deviation.

### Clinical results

No complications or side effects (e.g. hematoma, infection, allergic reaction, secondary arthritis) were observed. The mean range of motion, which was 129° before the first injection and 140° before the fifth injection, was significantly improved by the IA HA treatments (*P <* 0.001). The mean AKS knee score, which was 63 points before the first injection and 79 points before the fifth injection, was significantly improved by the IA HA treatments (*P <* 0.001). The mean AKS function score, which was 72 points before the first injection and 92 points before the fifth injection, was significantly improved by the IA HA treatments (*P <* 0.001). The mean values of all KOOS subscale scores and total KOOS score were significantly improved by the IA HA treatments (*P <* 0.001) (Table 2).

**Table 2:** **Changes in clinical results before the first HA injection (week 0) and fifth injection (week 5)**

	**Week 0**	**Week 5**	***P*** **value**
Range of motion (degrees)	129 ± 13	140 ± 7	<0.001
AKSKS (points)	63.8 ± 10.8	80.0 ± 10.1	<0.001
AKSFS (points)	72.6 ± 11.8	92.7 ± 7.0	<0.001
KOOS Pain (points)	56.2 ± 17.5	81.3 ± 11.2	<0.001
KOOS Symptoms (points)	62.6 ± 14.8	82.4 ± 12.8	<0.001
KOOS Activity of Daily Living (points)	72.6 ± 17.4	90.3 ± 8.1	<0.001
KOOS Sports (points)	45.1 ± 18.6	73.9 ± 13.6	<0.001
KOOS Quality of Life (points)	35.5 ± 14.8	63.6 ± 16.3	<0.001
Total KOOS score	272.0 ± 73.7	391.6 ± 50.8	<0.001

AKSKS: American Knee Society knee score. AKSFS: American Knee Society function score. KOOS: Knee injury and Osteoarthritis Outcome Score.

Data are given as mean ± standard deviation.

### Correlations among the biomarkers and the clinical scores

**Figure 2:**
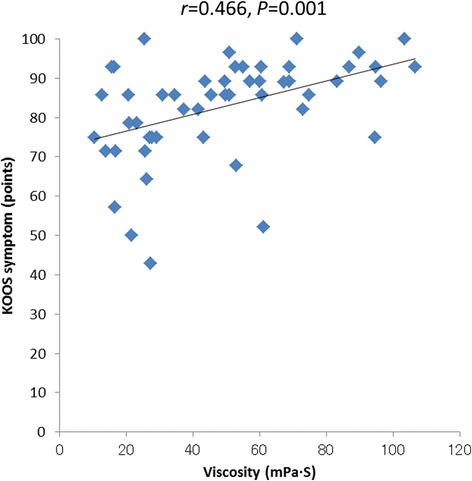
**Correlation between the SF viscosity measurement and the KOOS symptom score before the fifth IA HA treatment.**

**Figure 3:**
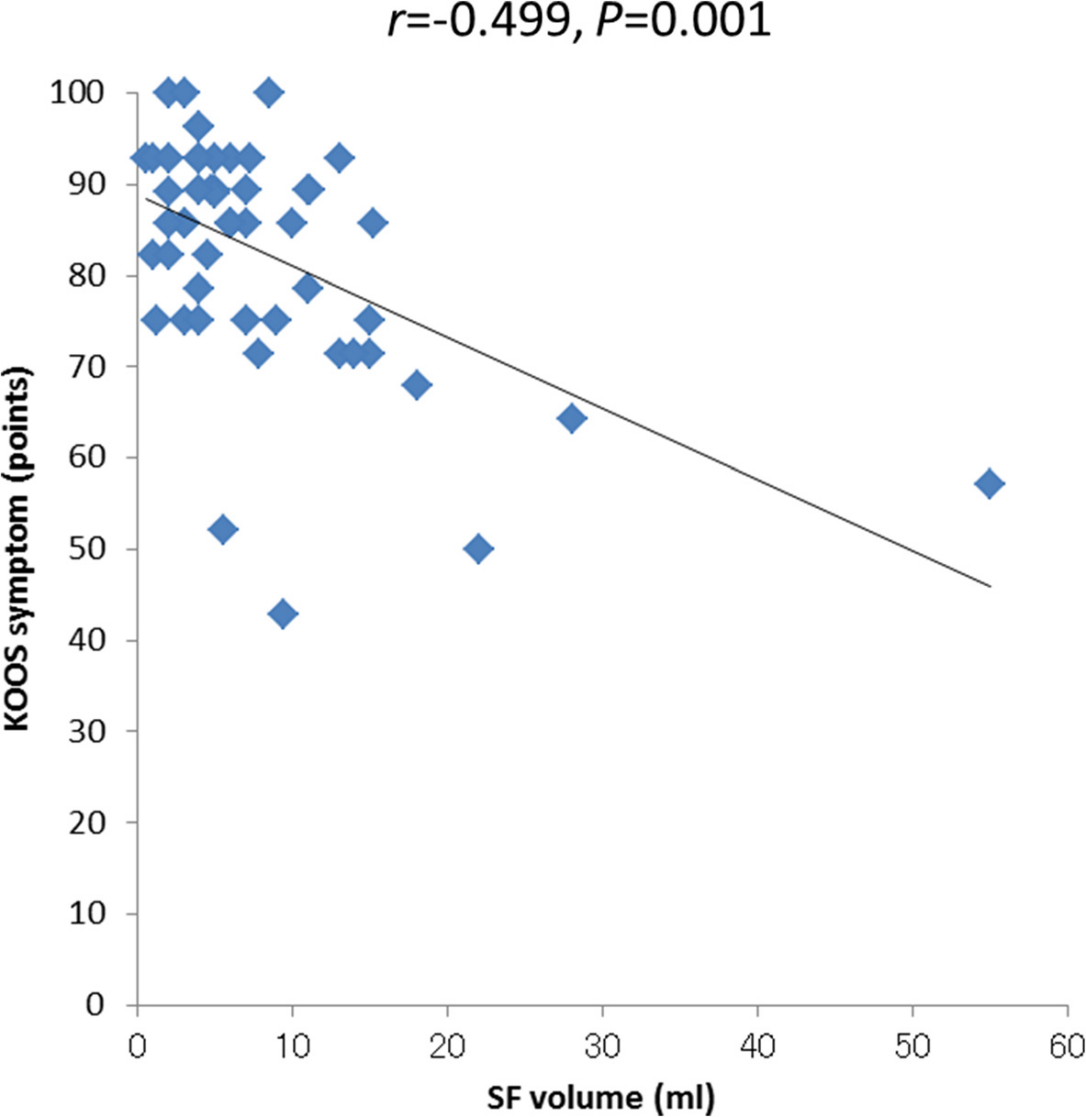
**Correlation between the SF volume measurement and the KOOS symptom score before the fifth IA HA treatment.**

Correlation analyses were performed between the biomarkers (HA, C6S, C4S, KS, IL-6, SF viscosity) and SF volume and the clinical scores before the first and fifth injections. Significant positive correlations were observed between the SF viscosity measurement and the KOOS symptom score (*r =* 0.466, *P =* 0.001) before the fifth injection (Figure 2). A significant negative correlation was observed between the SF volume measurement and the KOOS symptom score (*r =* −0.449, *P =* 0.001) before the fifth injection (Figure 3). No significant correlations were found between the other biomarkers and the clinical scores.

## Discussion

This study showed that biomarkers (C4S, C6S, IL-6) and the SF volume were significantly decreased and HA and viscosity were significantly increased after IA HA treatment. These improvements of SF properties indicated that IA HA had potential of affecting for the joint cartilage and synovium membrane with knee OA.

The factors that determine the development of knee OA are degeneration, abrasion, and destruction of the cartilage, synovitis associated with these cartilage changes, and further joint destruction resulting from decreased viscosity in the SF [1-3]. HA was reported to have inhibitory effects on cartilage destruction, anti-inflammatory effects, and supplementary effects for SF viscosity when used to treat these pathological conditions in OA models in vitro [39-42]. The present study showed that IA HA treatments can exert these three effects clinically by evaluating three types of joint markers.

IA HA caused significant decreases in C6S and C4S, which were used as biomarkers for cartilage degeneration and destruction. CS is bound to the mucopolysaccharide side chains of proteoglycans, which are the main components of joint cartilage [43]. Degeneration and destruction of joint cartilage leads to the release of CS into the SF [15,16]. CS is also related to the amount of articular cartilage, and a previous report described that CS in SF increases at the early stage of OA and decreases at the advanced stage of OA [44]. HA inhibits cartilage degeneration in vitro, as shown in studies using rabbits [42]. Our subjects were K/L 2 and 3, and they had residual cartilage. The results of C4S and C6S before the 1st injection were influenced by the acceleration of cartilage degeneration. The decrease of C4S and C6S between before the 1st and 5th injection did not indicate that residual cartilage was decreased by progression of OA, but that IA HA suppressed the degeneration of OA. C6S mainly consists joint cartilage and C4S is distributed not only cartilage, but also synovium membrane [15], the decrease of C4S and C6S may have the meaning which IA HA worked for both cartilage and synovium membrane. Some studies investigated the changes in CS after IA HA. Kobayashi et al. [45] showed that C6S and C4S were significantly decreased after IA HA (molecular weight: 800 kDa) treatments conducted for 16 patients with knee OA. Similarly, Hasegawa et al. [46] showed that C6S and C4S were significantly decreased after IA HA (molecular weight: 900 kDa) treatments conducted for 28 patients with knee OA. Meanwhile, Sugimoto et al. [47] showed that there were no significant changes in C6S and C4S after IA HA (molecular weight: 900 kDa) treatments conducted for 32 patients with knee OA. These findings demonstrate that there is no consensus about the CS changes after IA HA. Our study, which involved a larger sample size than the other studies, revealed significant decreases in C4S and C6S after IA HA treatments. The decreasing changes in these biomarkers support the possibility which IA HA have inhibitory effects of cartilage degeneration and releasing proteoglycans.

Campion et al. [16] reported that KS is useful biomarker for OA. Hasegawa et al. [46 ] showed that KS was significantly decreased after 5 weekly IA HA treatments conducted 28 patients with knee OA. We also thought to be useful for measurement of cartilage degeneration and measured KS. However, we were not able to show the significant change of KS. We considered that no significant change of KS also had a meaning. Biomarkers changes in the SF were not simply attenuated by repeated aspiration from the results of no significant change of KS and the decreasing of C4S, C6S and IL-6. The result of no change of KS is important to show no attenuation effect.

OA is known to cause inflammatory conditions and secondary synovitis [19]. IL-1, IL-6, tumor necrosis factor-alpha, and prostaglandin E2 have been reported to act as inflammatory biomarkers for OA [48-50]. Beekhuizen et al. [25] compared the levels of 47 cytokines in OA patients with those in healthy individuals and found that IL-6 was significantly increased. Similarly, Livshits et al. [26] found that IL-6 was a significant predictor of radiographic knee OA. IL-6 has various physiological effects, and causes destruction of cartilage via its angiogenic effects and activation of osteoclasts in joints [51,52]. Suppression of IL-6 has the possibility to be effective in protecting against the cartilage degeneration in OA. Therefore, the levels of IL-6 represent an indicator of the inflammatory conditions caused by OA and secondary synovitis, and were found to be significantly decreased after IA HA treatments in the present study. There are some previous reports about the levels of IL-6 in SF after IA HA treatment. Bianchi et al. [53] reported that oral treatment with non-steroidal anti-inflammatory drugs reduced the levels of IL-6 in SF, and Gallelli et al. [54] showed similar results. However, the changing levels of IL-6 in SF after IA HA treatment were not clear. HA was reported to act on the synovial membrane and inhibit synovitis in dogs [41]. In the present study, the decreased levels of IL-6 after IA HA treatments support our hypothesis that IA HA has inhibitory effects on synovitis and inflammatory reactions. Synovitis increases the SF volume, and we observed a significant decrease in the SF volume after IA HA. Our results support the opinion that HA has anti-inflammatory effects.

The levels of HA and viscosity, which are decreased in SF of patients with OA, are indicators of the therapeutic effects, and our results revealed significant increases in both factors after IA HA treatments. HA is present in the intra-articular SF, cartilage, and synovial membrane. The viscosity and water-holding capacity of HA in SF are responsible for its function as a shock absorber for the joint and provide lubrication to the joint [3]. HA infiltrates the synovial membrane and cartilage in vitro and promotes HA synthesis [41]. There are a few reports regarding the use of HA in actual clinical practice, which showed that HA supplementation through IA HA treatment increases viscosity and HA in SF [28,29,55]. The elimination half-life of HA in the knee joints of rabbits was reported to be approximately 12 hours, and injected HA may not remain in the knee joint beyond 1 week [56]. Contrary to these results, HA in SF was found to be increased after IA HA in this study. Accordingly, we believe that the increased HA reflects not only the injected HA but also newly produced HA, which resulted from improvement in the condition of the synovial membrane and cartilage caused by IA HA. Since the increase in SF viscosity is dependent on the HA level, the significant increase in HA would improve the properties of the SF after IA HA.

The relationships between biomarkers and clinical scores might enable us to identify the biomarkers that act as prognostic factors and indicate therapeutic effects. To date, researchers have reported several correlations between biomarkers and clinical scores. Hasegawa et al. [46] found a negative correlation between C4S or tenascin-C and VAS scores, while Sugimoto et al. [47] found a positive relationship between C6S or aggrecan and the JOA score. However, there are also reports that no correlations were found between biomarkers and clinical scores [45,55]. In the present study, we investigated the correlations between C6S, C4S, HA, IL-6, SF viscosity, or SF volume and each item in the AKS and KOOS scores. Our results indicated a positive correlation between SF viscosity and KOOS symptom scores before the fifth injection. No other positive correlations between biomarkers and clinical scores were observed. These results suggest that SF viscosity is related to the clinical scores. A higher viscosity of SF, which reflects the theoretical basis of IA HA, was found to be the important factor for the clinical scores. Additionally, the SF volume exhibited a negative correlation with the KOOS symptom scores at the fifth visit after IA HA treatment. No other negative correlations between biomarkers and clinical scores were observed. Increased SF volume elevated pressure in the knee joint, worsened range of motion and clinical symptom. The correlation coefficients (r) between viscosity and KOOS symptom, and SF volume and KOOS symptom were 0.466 and −0.499. These correlation coefficients might not be strong. However, these correlations indicate that viscosity and hydrarthrosis are important parameters for IA HA treatment.

Significant improvements were observed in the five subscales of the KOOS, AKS knee, and AKS function scores. The KOOS is a patient-based clinical assessment score, for which the patients themselves input their subjective symptoms. Meanwhile, the AKS score is a physician-based clinical assessment score, in which a physician examines the patient, takes a history, and evaluates the symptoms. Improvements were observed in both the patient-based KOOS and physician-based AKS scores in the present study. The measurements of two different types of clinical score were more reliable in researching for efficacy of IA HA.

This study has several limitations. First, the study did not have a control group, because our hospital decided that it was ethically inappropriate to create such a control group. Without control group, we were not able to deny the possibility of attenuation effect by repeated aspiration. Second, the follow-up period was short. Although there were significant changes in joint biomarkers after IA HA treatments, the results of long-term treatment remain unclear. Third, many patients in this study had early and moderate OA (K/L grade 2 and 3). This is because patients with terminal OA (K/L grade 4) are indicated for surgical treatment rather than conservative treatment. Therefore, there may have been patient bias. Fourth, our results showed biomarkers of C4S, C6S, KS and IL-6. We were not able to mention other biomarkers in the SF.

## Conclusion

To the best of our knowledge, this is the first report of simultaneous measurements of HA levels together with SF viscosity and levels of biomarkers C6S, C4S, KS, and IL-6 to evaluate the efficacy of IA HA before first and fifth injection. Also, all of the clinical symptoms were significantly improved. The changes in the levels of biomarkers suggested that IA HA treatments may have cartilage-protective effects, anti-inflammatory effects, and SF viscosity-replenishing effects.
